# Global Trends in Nursing-Related Research on COVID-19: A Bibliometric Analysis

**DOI:** 10.3389/fpubh.2022.933555

**Published:** 2022-07-18

**Authors:** Qian Zhang, Shenmei Li, Jing Liu, Jia Chen

**Affiliations:** ^1^Xiangya Hospital Department of Neurosurgery, Central South University, Changsha, China; ^2^Department of Nursing, Guilin Medical University, Guilin, China; ^3^Xiangya Nursing School, Central South University, Changsha, China

**Keywords:** COVID-19, nursing, bibliometric analysis, hotspots, CiteSpace, VOSviewer

## Abstract

**Background:**

Coronavirus disease 2019 (COVID-19) has significantly impacted healthcare, especially the nursing field. This study aims to explore the current status and hot topics of nursing-related research on COVID-19 using bibliometric analysis.

**Methods:**

Between 2019 and 2022, publications regarding nursing and COVID-19 were retrieved from the Web of Science core collection. We conducted an advanced search using the following search query string: TS = (“Novel coronavirus 2019” or “Coronavirus disease 2019” or “COVID 19” or “2019-nCOV” or “SARS-CoV-2” or “coronavirus-2”) and TS = (“nursing” or “nurse” or “nursing-care” or “midwife”). Bibliometric parameters were extracted, and Microsoft Excel 2010 and VOSviewer were utilized to identify the largest contributors, including prolific authors, institutions, countries, and journals. VOSviewer and CiteSpace were used to analyze the knowledge network, collaborative maps, hotspots, and trends in this field.

**Results:**

A total of 5,267 papers were published between 2020 and 2022. The findings are as follows: the USA, China, and the UK are the top three prolific countries; the University of Toronto, the Harvard Medical School, the Johns Hopkins University, and the Huazhong University of Science & Technology are the top four most productive institutions; Gravenstein, Stefan, and White, Elizabeth M. from Brown University (USA) are the most prolific authors; The International Journal of Environmental Research and Public Health is the most productive journal; “COVID-19,” “SARS-CoV-2,” “nurse,” “mental health,” “nursing home,” “nursing education,” “telemedicine,” “vaccine-related issues” are the central topics in the past 2 years.

**Conclusion:**

Nursing-related research on COVID-19 has gained considerable attention worldwide. In 2020, the major hot topics included “SARS-CoV-2,” “knowledge,” “information teaching,” “mental health,” “psychological problems,” and “nursing home.” In 2021 and 2022, researchers were also interested in topics such as “nursing students,” “telemedicine,” and “vaccine-related issues,” which require further investigation.

## Introduction

In late 2019, the first Coronavirus disease 2019 (COVID-19) case was reported in Wuhan, China, which has eventually spread worldwide (1). On January 30, 2020, the World Health Organization declared the outbreak to be an international emergency (2). Facing this global challenge for humanity, healthcare workers are first on the front line. Nurses play a pivotal role in controlling, mitigating infection, and providing primary and intensive care (3) despite putting their own lives at risk while combating COVID-19 (4). In Italy and Spain, the percentage of healthcare workers infected by COVID-19 was 20% (5, 6). Nurses were at high risk of being infected by COVID-19 because they were in close contact with patients and were exposed to them for a long time. According to the International Council of Nurses, the death due to COVID-19 among health professionals accounts for 7% of the total deaths, much higher than the general population (7). Liu et al. reported that nurses had experienced tremendous stress because of the stigmatization, increased risk of infection and mortality, heavy workload, and lack of protective gear and staff, in addition to the challenges related to providing care for patients during the COVID-19 pandemic (8). Besides, Chew et al. reported that among healthcare workers caring for COVID-19 patients, 8.7% showed moderate to extremely severe anxiety, 5.3% showed moderate to very severe depression, and 2.2% showed moderate to extremely severe stress (9). In addition, due to the pandemic and lockdown, nursing students have faced additional challenges, such as economic uncertainty, concern about infection, and difficulties with online learning during the COVID-19 pandemic (10, 11). The outbreak of COVID-19 has further emphasized the importance of nursing discipline in public health. Numerous studies on nursing and COVID-19 have been published in the past 2.5 years. However, no study has comprehensively analyzed the profile of nursing-related research on COVID-19 and presented potential future research directions in this area.

Bibliometric analysis is a quantitative technique that applies bibliometric tools (e.g., CiteSpace, VOSviewer, Bibliometrix R, Pajek, and Gephi) to analyze the scientific knowledge network and evolution in a given field. It summarizes the publication trend, the highest citation articles, leading researchers, institutions, countries, journals, and cooperation. The technique also allows for the detection of valuable references and visualization of hot topics and potential research directions in a particular field (12). Thus, this study aims to elucidate the knowledge structure and the main topics in nursing research on COVID-19 through a bibliometric analysis. Several directions for future research based on the findings are also presented in this paper.

## Methods and Materials

### Search Strategy

On March 24, 2022, an advanced search was conducted on WoSCC using the search query string, TS = (“Novel coronavirus 2019” or “Coronavirus disease 2019” or “COVID 19” or “2019-nCOV” or “SARS-CoV-2” or “coronavirus-2”) and TS = (“nursing” or “nurse” or “nursing-care” or “midwife”), to identify publications related to COVID-19 in nursing research. The document type was restricted to articles and reviews, and the language was limited to English. The database was searched and screened independently by Jing Liu and Qian Zhang. Discrepancies were resolved through discussions with the other two authors (SL and JC) until a consensus was reached.

### Data Extraction and Analytical Methods

Bibliometric parameters were extracted (e.g., title, keywords, authors, institutions, countries or regions, journal, publication year, total citations (TC), citations per publication (CPP), and cited references) and exported to the Microsoft Excel 2010 (Redmond, Washington, USA) and VOSviewer (version 1.6.11, Leiden University, Leiden, Netherlands) to identify the largest contributors, including prolific authors, institutions, and countries. VOSviewer and CiteSpace (Version 5.8. R3) were used to illustrate the map and strength of the collaboration between authors, institutions, and countries to demonstrate their influence in nursing research on COVID-19. In addition, keyword bursts and reference bursts were used to capture the knowledge base in this field. Furthermore, the co-occurrence of author keywords in VOSviewer and keyword co-occurrence in CiteSpace were utilized to visualize the hot topics and demonstrate the potential research frontiers. In the map of VOSviewer and CiteSpace, the node size represents the number of publications, whereas the line indicates the links between them. The larger the node, the higher the number of publications, while the thicker the line, the stronger the cooperation between the two nodes (13).

## Results

### General Data

**Figure 1:**
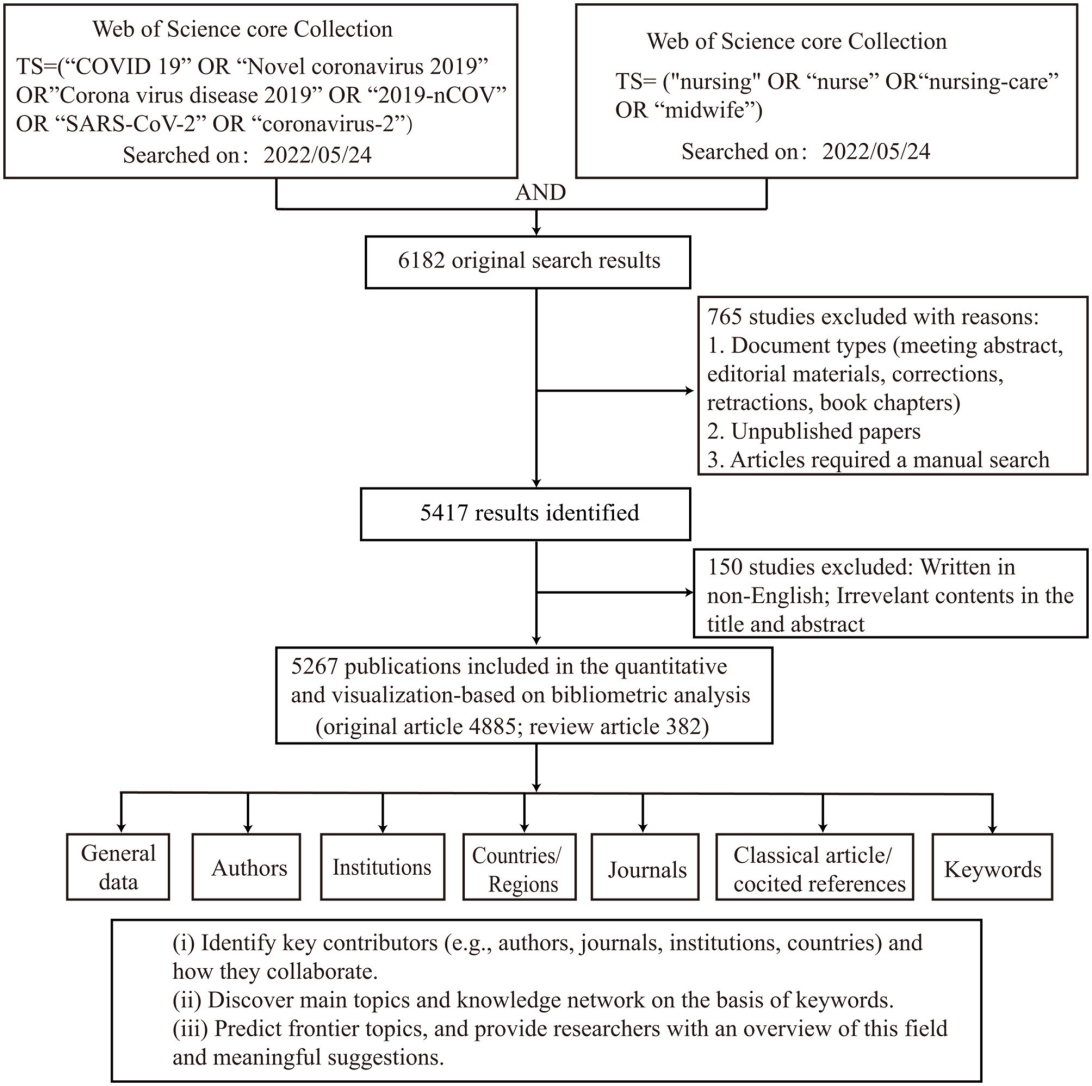
Flow chart of data screening and bibliometric analysis.

**Figure 2:**
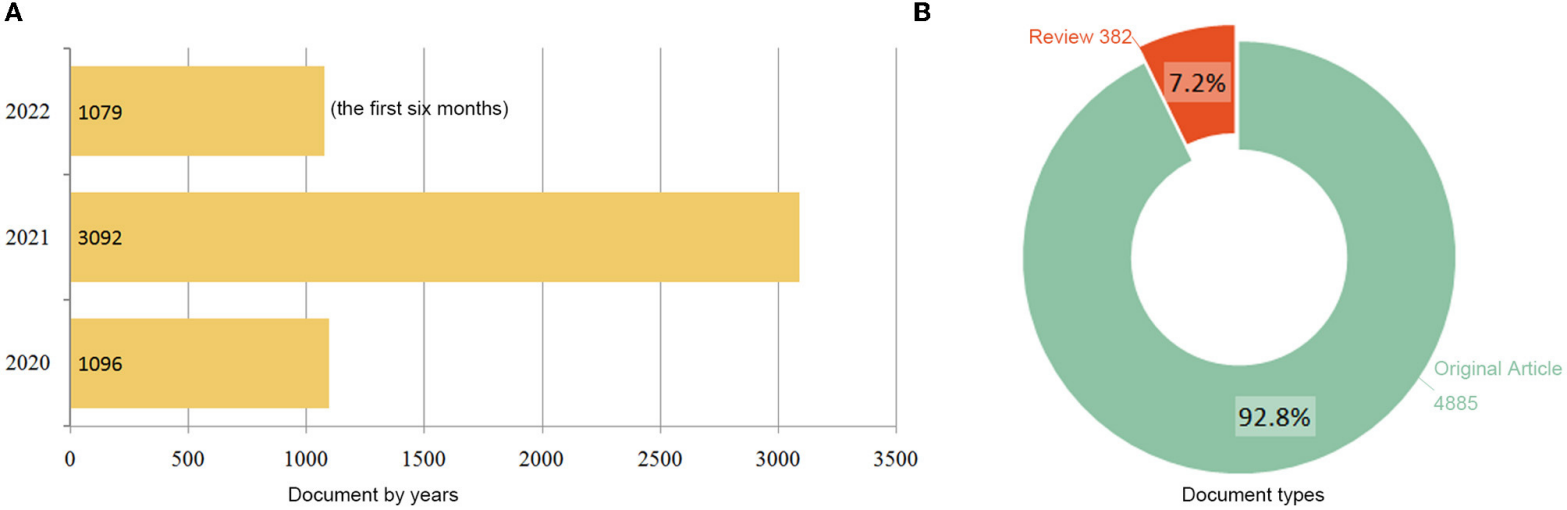
Distribution of publications by year **(A)** and type **(B)**.

The study design and analytic approach are presented in Figure 1. The initial search query returned 6,182 results. After restricting the type of literature (original research and review) and the English language, 5,267 articles were retrieved. The following parameters were also determined: 55,224 TC; 10.48 CPP; 85 H-index. In total, 29,190 authors, 7,926 institutions, 134 countries/territories, and 1,144 journals were involved in these publications. As shown in Figure 2, the number of publications has increased from 1,096 in 2020 to 3,092 in 2021; a total of 1,079 publications were recorded in the first six months of 2022. Original articles constitute 92.8% of retrieved publications, while the remaining 7.2% are review articles.

### Top Contributing Authors

**Figure 3:**
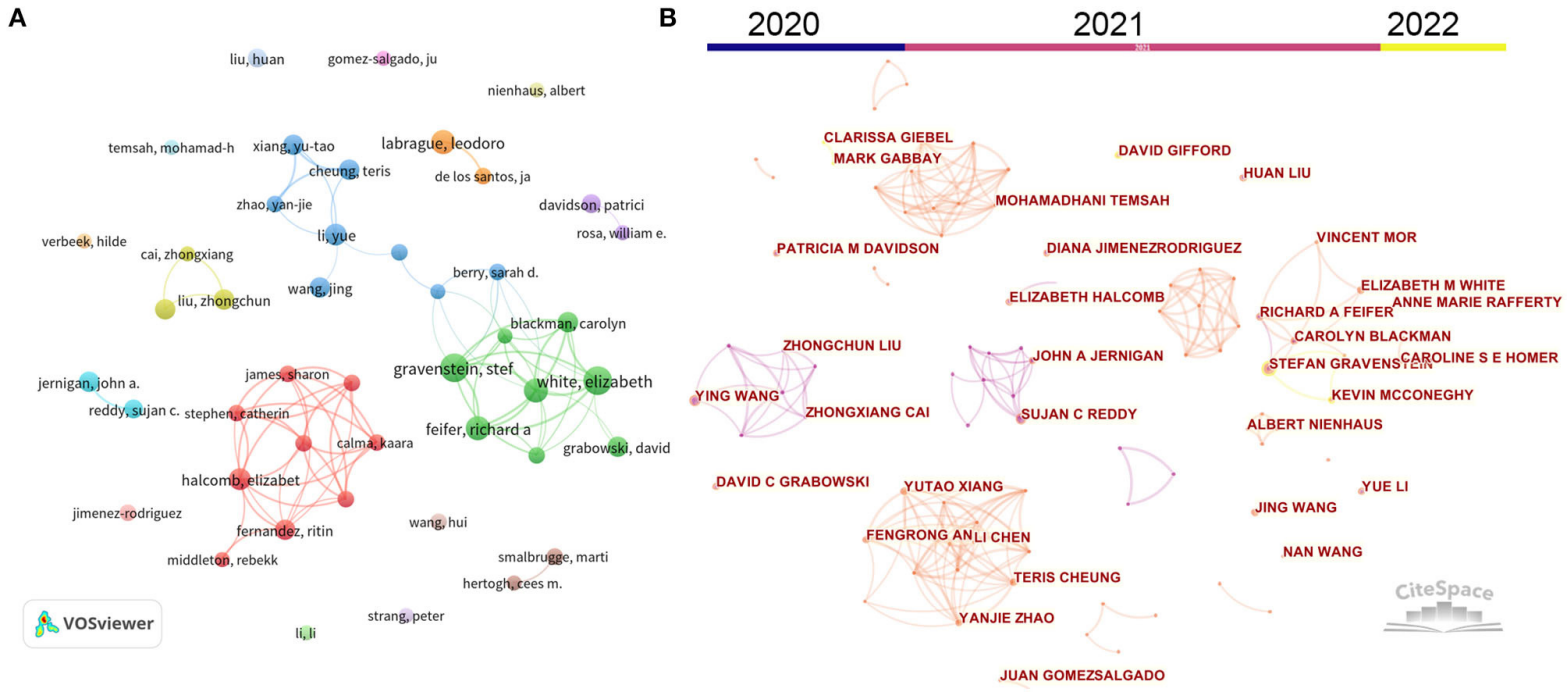
The cooperation network between the most productive authors based on VOSviewer **(A)** and CiteSpace **(B)**. Node size indicates the number of publications. The width of links refers to the cooperation strength.

Table 1 presents the top contributing authors in nursing-related research on COVID-19. Gravenstein, S and White, EM from Brown University (USA) were identified as the most prolific author with 15 publications. They are followed by Labrague, LJ (Sultan Qaboos University, Oman), Feifer, RA (Genesis Health Care, USA), and Mor, V (Brown University, Australia) with 12 publications. However, the author with the highest citations was identified as Liu, ZC from Wuhan University, China (3,934 TC and 393.4 CPP), followed by Wang, Y from Wuhan University, China (3,500 TC and 350 CPP) and Cai, ZX from Wuhan University, China (3,472 TC and 496 CPP). The author's cooperation network was analyzed by VOSviewer and CiteSpace. As shown in Figure 3A, 45 authors with more than seven publications were identified by VOSviewer. Similarly, these authors were also identified by CiteSpace, as shown in Figure 3B, which also displayed their respective active periods. Reddy, SC, Jernigan, JA, Liu, ZC, and Wang, Y were active in 2020, while Gravenstein, S, White, EM, Feifer, RA, and Xiang, YT were active in 2021; Gravenstein, S, Gifford, D, and Mcconeghy, K were active in 2022. Six scholar groups with numerous collaborations were determined, and more collaborations occurred within the group comprised Jernigan, JA and Reddy, SC, and within the group comprised Gravenstein, S and White, EM.

**Table 1:** Top 15 most prolific authors.

**Rank**	**Author**	**Publication**	**TC**	**CPP**	**Institution**	**Country**
1a	Gravenstein, Stefan	15	190	12.7	Brown University	USA
1b	White, Elizabeth M.	15	251	16.7	Brown University	USA
2a	Labrague, Leodoro J.	12	485	40.4	Sultan Qaboos University	Oman
2b	Feifer, Richard A.	12	190	15.8	Genesis HealthCare	USA
2c	Mor, Vincent	12	181	15.1	Brown University	USA
3a	Halcomb, Elizabeth	11	310	28.2	University of Wollongong	Australia
3b	Yue Li	11	229	20.8	University of Rochester	USA
4a	Blackman, Carolyn	10	168	16.8	Genesis Health Care	USA
4b	Fernandez, Ritin	10	277	27.7	Hong Kong Polytechnic University	China
4c	Grabowski, David C.	10	406	40.6	Harvard Medical School	USA
4d	Jernigan, John A.	10	252	25.2	Centers for Disease Control & Prevention	USA
4e	Zhongchun Liu	10	3,934	393.4	Wuhan University	China
4f	Jing Wang	10	92	9.2	Huazhong Univ Sci & Technol	China
4g	Ying Wang	10	3,500	350.0	Wuhan University	China
4h	Yu-Tao Xiang	10	113	11.3	University of Macau	China

*TC, total citations; CPP, citations per publication*.

### Top Contributing Institutions

**Figure 4:**
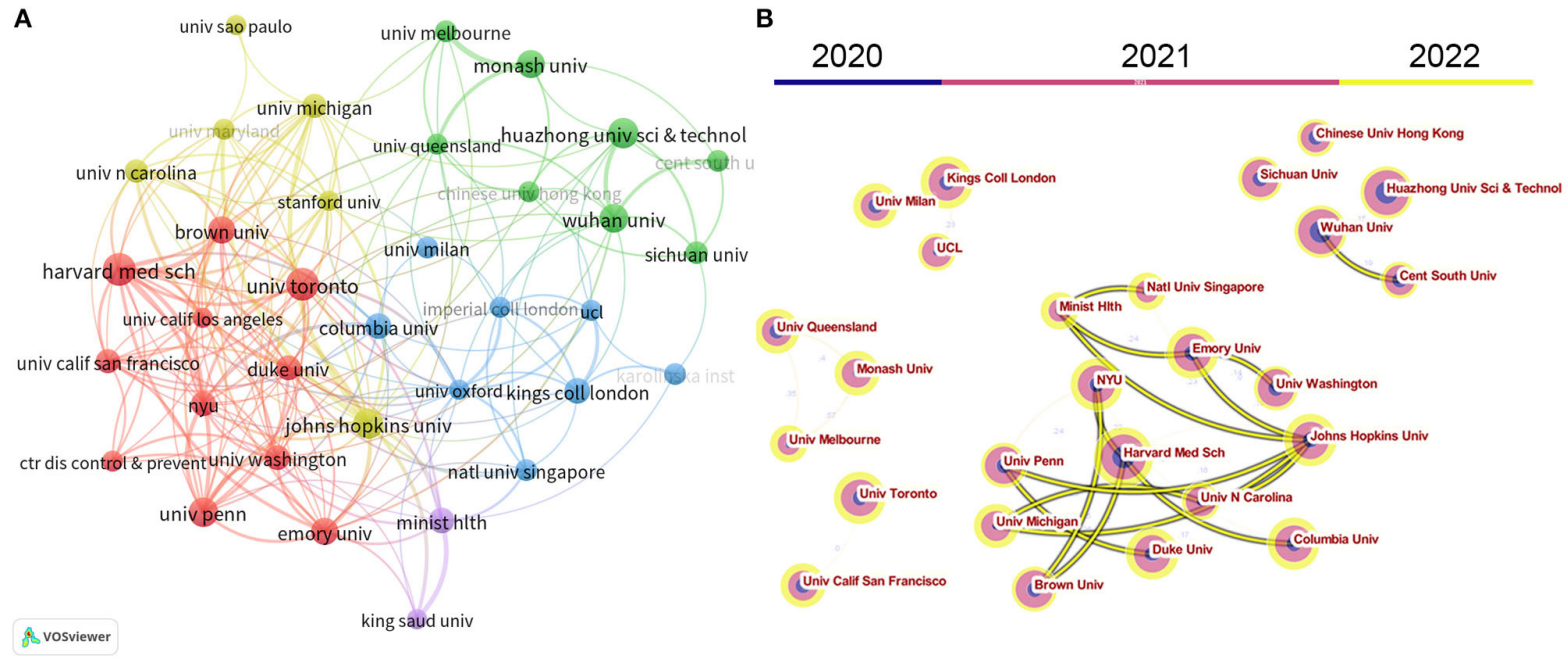
The cooperation network between institutions based on VOSviewer **(A)** and CiteSpace **(B)**. Node size indicates the number of publications. The link size refers to the cooperation Intensity.

Table 2 lists the most prolific institutions identified in this study. The top 10 contributing institutions have published 11.6% of articles in this field. The University of Toronto (Canada) ranks first with 76 publications, followed by the Harvard Medical School (USA) with 75 publications, the Johns Hopkins University (USA) with 67 publications, and the Huazhong University of Science & Technology (China) with 61 publications. In terms of the number of citations, the Wuhan University (China) ranks first with 7,233 TC, followed by the University of Washington (USA) with 2,471 TC, and the Center for Disease Control & Prevention (USA) with 2,050 TC. The institution's cooperative network was visualized by VOSviewer and CiteSpace. As shown in Figure 4A, 35 institutions with at least 30 publications were identified by VOSviewer, and Figure 4B presents the time evolution of these institutions. Institutions in China were active in 2020 and 2021 but less active in 2022 (e.g., Huazhong University of Science & Technology, Wuhan University, and Central South University); Institutions in North America, European, and Australia were active in 2021 and especially, 2022. The results demonstrate that inter-institutional cooperation exhibits a typical regional character and primarily occurs in the same country.

**Table 2:** Top 10 prolific institutions.

**Rank**	**Institution**	**Publication**	**TC**	**CPP**	**Country**
1	Univ Toronto	76	568	7.5	Canada
2	Harvard Med Sch	75	1,334	17.8	USA
3	Johns Hopkins Univ	67	625	9.3	USA
4	Huazhong Univ Sci & Technol	65	1,919	29.5	China
5	Univ Penn	61	875	14.3	USA
6	Wuhan Univ	58	7,233	124.7	China
7	Monash Univ	56	438	7.8	Australia
8	Brown Univ	54	890	16.5	USA
9	Emory Univ	54	321	5.9	USA
10	Kings Coll London	48	1,549	32.3	UK

*TC, total citations; CPP, citations per publication*.

### Top Contributing Countries

**Figure 5:**
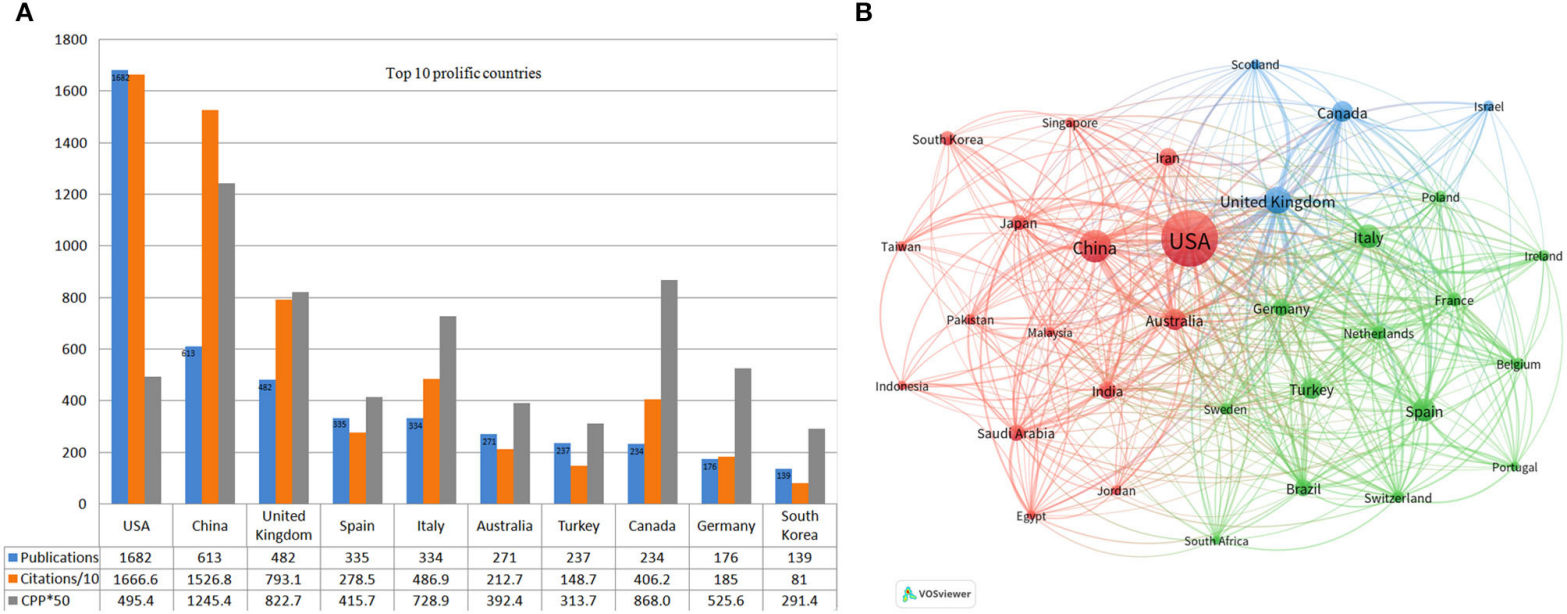
**(A)** Top 10 prolific countries, the number of publications, total citations (× 0.1), and citations per publication (× 50) for each country. **(B)** Collaboration among countries. Node size indicates the number of articles. The width of links indicates the cooperation strength.

Figure 5 displays the top productive countries and their respective collaboration. The study found the USA as the most productive country with 1,682 publications (31.9% of the total) and 16,666 TC, followed by China (613 publications, 15,268 TC) and the UK (482 publications and 7,931 TC) (Figure 5A). In terms of CPP, the USA ranks sixth (*n* = 9.9), lower than China (*n* = 24.9), Canada (*n* = 17.4), the UK (*n* = 16.9), Italy (14.6), and Germany (10.5). Analysis of the co-authorship-country by VOSviewer revealed the cooperation between countries. With at least 50 publications, a total of 28 countries were selected for the visualization, of which the USA, China, the UK, Italy, Spain, and Australia are represented by the most prominent nodes with relatively thicker links, signifying their closer collaboration and academic influence in this area (Figure 5B).

### Top Contributing and Co-cited Journals

Table 3 lists the top 10 active journals publishing articles related to nursing research on COVID-19, which are ranked either Q1 or Q2 by JCR. At the top of the list is the International Journal of Environmental Research and Public Health (*n* = 285), followed by BMJ Open (*n* = 118) and the Journal of Nursing Management (*n* = 97). However, the Journal of Nursing Management has recorded the highest CPP (*n* = 12.2), followed by the Journal of the American Medical Directors Association (*n* = 11.2) and the Frontiers in Psychiatry (*n* = 10.4). In terms of co-citation, the New England Journal of Medicine ranks first (*n* = 2,883), followed by the Lancet (*n* = 2,833) and JAMA—Journal of the American Medical Association (*n* = 2,754).

**Table 3:** The top 10 prolific journals and co-cited journals.

**Rank**	**Journal**	**Publication**	**TC**	**CPP**	**IF**	**JCR**	**Co-cited journal**	**Co-citations**
1	Int J Env Res Pub He	285	1,651	5.8	3.389	Q1	New Engl J Med	2,883
2	BMJ Open	118	432	3.7	2.874	Q2	Lancet	2,833
3	J Nurs Manage	107	1,307	12.2	4.368	Q1	JAMA-J Am Med Assoc	2,754
4	Plos ONE	107	878	8.2	3.24	Q2	Int J Env Res Pub He	2,390
5	J Am Med Dir Assoc	97	1,087	11.2	6.462	Q2	PLoS ONE	1,947
6	Frontiers in Public Health	81	254	3.1	6.075	Q1	BMJ-Brit Med J	1,543
7	Journal of Advanced Nursing	78	296	3.8	2.662	Q1	J Clin Nurs	1,465
8	Journal of Clinical Nursing	78	742	9.5	3.058	Q1	J Am Geriatr Soc	1,404
9	Frontiers in Psychology	77	599	7.8	3.942	Q2	J Nurs Manage	1,352
10	Frontiers in Psychiatry	73	760	10.4	5.108	Q2	JAMA Netw Open	1,330

*TC, total citations; CPP, citations per publication; IF, impact factor (2021); JCR, Journal Citation Reports (2021)*.

### Top Cited Articles

Table 4 lists the top 10 most cited publications. Of which, five papers discuss the mental health of healthcare workers; three papers report the transmission of SARS-CoV-2 in nursing facilities and long-term care facilities; one article provides the guideline for COVID-19; one article explores the outcomes of COVID-19 patients 6 months after being discharged from the hospital. Lai, J et al. published an article with the highest citation count (2,828 TC) in JAMA Network Open. In this article, the authors investigated the psychological problems and associated risk factors among 1,257 healthcare workers who treat patients suspected or confirmed for COVID-19 in 39 hospitals in China (14). The second most cited article (2,049 TC) was produced by Jin et al. in Military Medical Research. The authors provide a guideline for researchers and policy makers based on their successful experience in treating severe COVID-19 cases (15). Pappa et al. produced a review article, which gained the third-highest citation count (1,195 TC) and was accepted by Brain Behavior and Immunity. The authors meta-analyzed 33,062 participants in 13 studies; they found that among healthcare workers working on the front line, 23.2% suffered from anxiety, 22.8% suffered from depression, and 38.9% suffered from insomnia. The article also reports that females and nurses are at a 5–10% higher risk of suffering these symptoms than males and other medical personnel (16).

**Table 4:** Top 10 cited articles.

**Rank**	**Title**	**First authors**	**Type**	**Citation**	**Journal**	**Year**
1	Factors associated with mental health outcomes among health care workers exposed to coronavirus disease 2019	Lai, JB	Article	2,828	JAMA Netw. Open	2020
2	A rapid advice guideline for the diagnosis and treatment of 2019 novel coronavirus (2019-nCoV) infected pneumonia (standard version)	Jin, YH	Article	2,049	Military Med. Res.	2020
3	Prevalence of depression, anxiety, and insomnia among healthcare workers during the COVID-19 pandemic: A systematic review and meta-analysis	Pappa, S	Review	1,195	Brain Behav. Immun.	2020
4	Presymptomatic SARS-CoV-2 Infections and Transmission in a Skilled Nursing Facility	Arons, MM	Article	1,125	N. Engl. J. Med.	2020
5	6-month consequences of COVID-19 in patients discharged from hospital: a cohort study	Huang, CL	Article	1,065	Lancet	2021
6	Psychiatric and neuropsychiatric presentations associated with severe coronavirus infections: a systematic review and meta-analysis with comparison to the COVID-19 pandemic	Rogers, JP	Review	783	Lancet Psychiatry	2020
7	Epidemiology of Covid-19 in a Long-Term Care Facility in King County, Washington	McMichael, TM	Article	713	N. Engl. J. Med.	2020
8	A multinational, multicentre study on the psychological outcomes and associated physical symptoms amongst healthcare workers during COVID-19 outbreak	Chew, NWS	Article	644	Brain Behav. Immun.	2020
9	Asymptomatic and presymptomatic SARS-CoV-2 infections in residents of a long-term care skilled nursing facility - King County, Washington, March 2020	Kimball, A	Article	630	MMWR-Morb. Mortal. Wkly. Rep.	2020
10	The psychological and mental impact of coronavirus disease 2019 (COVID-19) on medical staff and general public - A systematic review and meta-analysisy	Min Luo	Review	555	Psychiat Res	2020

### Analysis of Co-citation References

**Figure 6:**
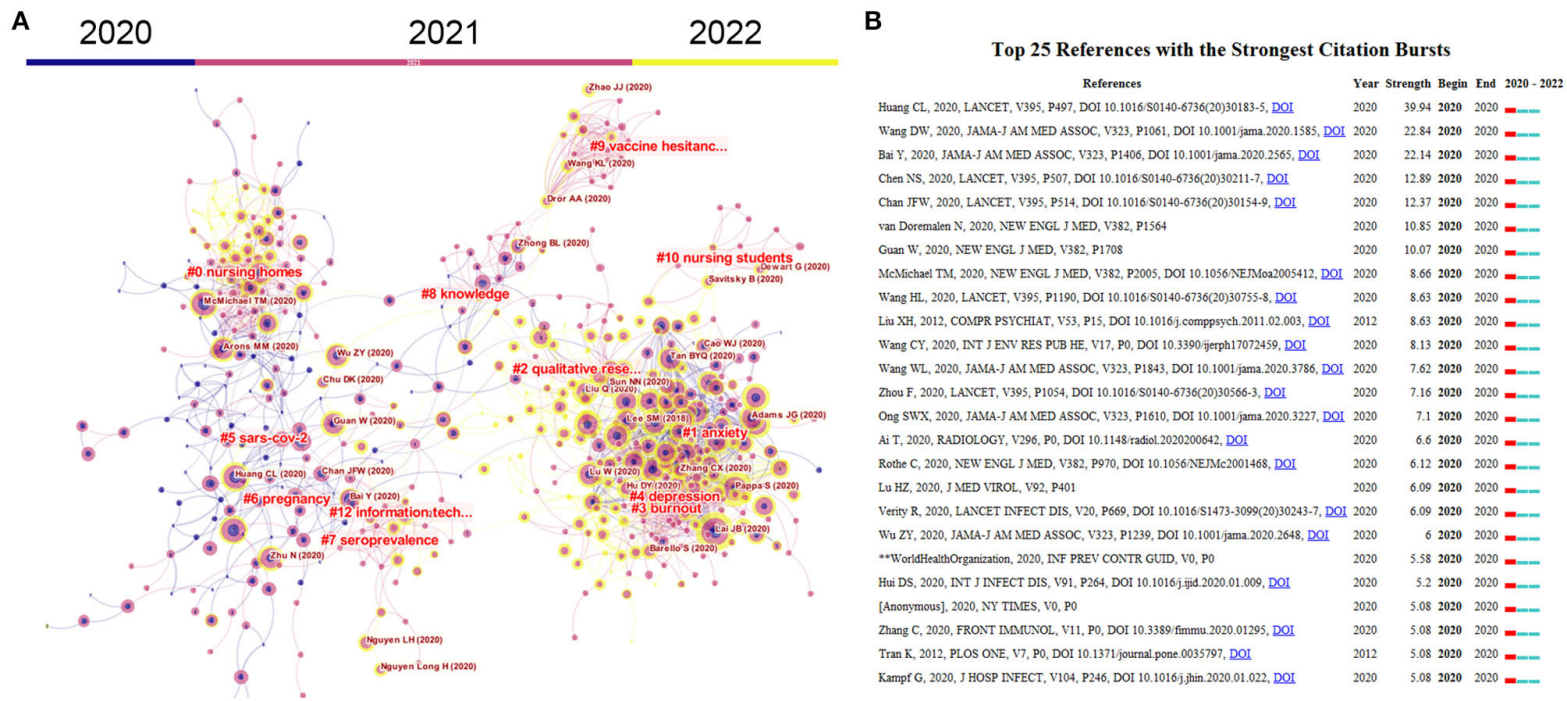
**(A)** Reference co-citation network clustered by CiteSpace. The nodes and links are distinguished by colors, in which dark color refers to an earlier co-citation relationship. References with at least 100 citations are displayed in the network in nodes named by the first author (publication year). The size of the node is positively associated with the citation number. The red writing reports the name of the cluster auto-identified by the Citespace LLR algorithm. **(B)** Top 25 references with the strongest bursts. The red bar indicates the burst duration. The burst strength suggests the importance of this article to the research field.

To discover the evolution of scientific paradigms in nursing research on COVID-19, we conducted a co-citation analysis using CiteSpace. As shown in Figure 6A, articles with at least 100 citations are displayed in the network. Articles published in 2020 were centered around the topics, namely “SARS-CoV-2,” “knowledge,” “information teaching,” “burnout,” “depression,” and “anxiety.” Topics, namely “nursing student” and “vaccine-related issues,” make up the largest discussion in articles published in 2021, representing the major concern at that time. In addition, a citation burst was used to identify significant reference contributing to this field's knowledge. The top 25 publications with the highest citation burst were identified by CiteSpace (Figure 6B). Huang, C et al. published the article with the highest citation bursts (*n* = 39.94) in the Lancet on January 4, 2020. In this article, the authors introduced the epidemiological, clinical, laboratory, and radiological characteristics of COVID-19 and summarized the treatment and clinical outcomes of 41 cases with SARS-CoV-2 infection in Wuhan, China (17). Wang, D et al. published the article with the second-highest citation bursts (*n* = 22.84) in JAMA on February 7, 2020, reporting their findings regarding the features of 138 patients with COVID-19 in Wuhan, China. The article highlights that 41% of patients infected with COVID-19 within the hospital and 26% of patients require intensive care (18). The article with the third-highest citation bursts was published by Bai, Y et al., which reports on five patients in a familial cohort in Anyang (China) who were infected with COVID-19 through contact with asymptomatic carriers (negative chest CT imaging and positive nasopharyngeal swabs). The article emphasizes the potential challenge in preventing COVID-19 infection associated with the asymptomatic carriers (19).

### Analysis of Keywords

**Figure 7:**
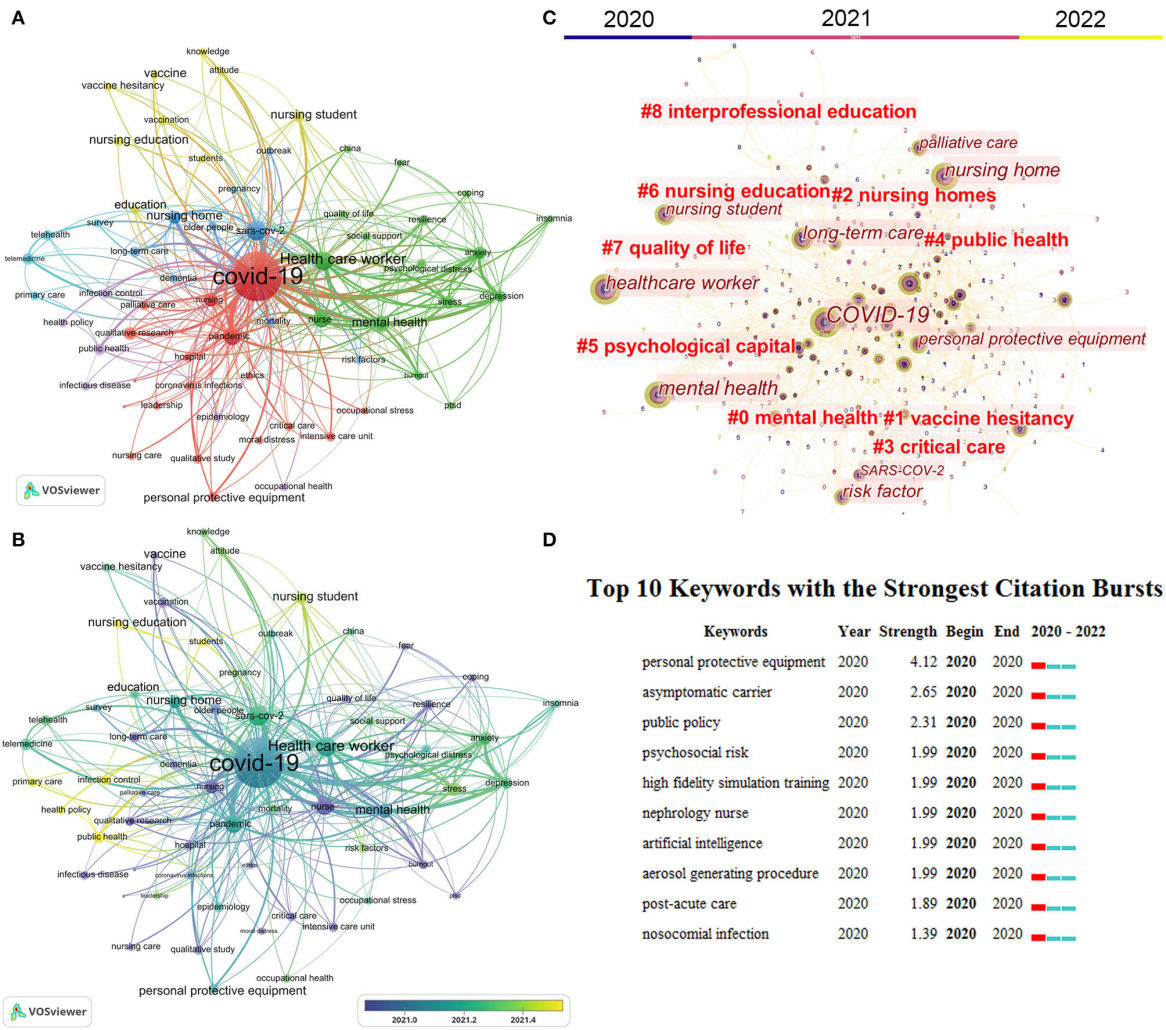
Analysis of author keywords. **(A)** The co-occurrence networks of keywords are visualized by VOSviewer. Large nodes represented keywords with high frequency; the same color indicates closer relationships; **(B)** chronological overview of the co-occurrence network of author keywords. Dark blue refers to an earlier appearance, and yellow refers to the latest appearance. **(C)** Keywords clusters named by the CiteSpace LLR algorithm from 2020 to 2022. **(D)** Top 10 keywords with the strongest citation bursts. The red bar indicates the burst duration. The burst strength refers to the importance of the keyword to the research field.

To present major themes and potential research trends in this field, we conducted a keyword co-occurrence analysis using VOSviewer and CiteSpace. In addition, we used a thesaurus (Supplementary Table S1) to merge these keywords with similar meanings. For example, coronavirus-2 was replaced by SARS-CoV-2 and Coronavirus disease 2019 was replaced by COVID-19. A total of 8,031 author keywords were identified by VOSviewer. The co-occurrence network was only visualized for the keywords that occurred more than 30 times. Finally, 63 keywords were classified into six clusters with different colors (Figure 7A). The top 10 keywords with highest number of occurrences are “COVID-19” (*n* = 3,172), “SARS-CoV-2” (*n* = 640), “health care worker” (*n* = 595), “pandemic” (*n* = 531), “nurse” (*n* = 487), “nursing” (*n* = 323), “mental health” (*n* = 323), “nursing home” (*n* = 310), “anxiety” (*n* = 291), and “depression” (*n* = 236).” Furthermore, to decipher potential research directions in this field, we used a timeline view of keyword co-occurrence analysis in VOSviewer and Citespace. As displayed in Figures 7B,C, keywords are colored according to their average publication years. Dark colors (e.g., blue and dark blue) indicate popular keywords in the early stages, namely “nursing,” “nurse,” “burnout,” “PTSD,” “fear,” “coping,” “intensive care unit,” “critical care,” “personal protective equipment,” “nursing home,” “older people,” “long-term care,” “vaccine,” “vaccination,” “telemedicine,” “telehealth.” Light colors (e.g., yellow-green and yellow) represent the recent popular keywords, namely “occupational health,” “stress,” “depression,” “anxiety,” “nursing student,” “nursing education,” “public health,” “public policy,” “infection control,” and “vaccine hesitancy”. The burst module in CiteSpace allows for identifying keywords frequently used during a particular period (20). The top 10 keywords with the strongest citation bursts are displayed in Figure 7D, which shows that “personal protective equipment” is the keyword with the highest burst strength (*n* = 4.12), followed by “asymptomatic carriers” (*n* = 2.65) and “public policy” (*n* = 2.31).

## Discussion

The global outbreak of COVID-19 has been considered a severe risk for healthcare providers, especially nurses (21). As a result, numerous articles related to the issue were published during the COVID-19 pandemic, which is evident in the sharp rise in its growth rate. The number of papers published in 2021 was nearly 3-fold that in 2020, and by the first 6 months of 2022, 1,079 articles were published. *In-vivo* and *in-vitro* studies show that with the new variants emerging (Delta and Omicron), the pathogenicity of SARS-CoV-2 has become milder than that of the ancestral strain (22, 23). Several countries have no longer implemented strict health policies to prevent and control COVID-19. For example, British Prime Minister Boris Johnson announced that from February 24, all COVID-19 prevention and control measures in Britain would be suspended and launched the “Living with COVID-19” program. Nonetheless, the COVID-19 pandemic may have a lasting impact on society, science, and education. Thus, it is expected that there will be more publications describing the impact of COVID-19 on the nursing field.

### Author Analysis

Gravenstein, S and White, EM from Brown University (USA) were identified as the most prolific author (*n* = 15 publications) in this study. In terms of TC and CPP, Liu, ZC (3,934 TC and 393.4 CPP) and Wang, Y (3,500 TC and 350 CPP) from Wuhan University (China) rank first and second, respectively. This finding shows that by referring to the works of these authors, new researchers can gain an idea about designing impactful research and grasp hot topics in the field. For example, in 2020, Gravenstein, S and White, EM mainly focused on the characteristics of COVID-19 transmission in nursing homes and skilled nursing facilities (24, 25), and in 2021 and 2022, the focus shifted to the risk factors (26), treatment (27), and the effect of vaccination on health workers and residents in a nursing home (28, 29). The majority of articles (8/10) by Liu, ZC and Wang, Y were published in 2020, focusing on the impact of COVID-19 on the mental health and psychological state of healthcare workers (14, 30, 31).

### Country and Institution Analysis

A total of 134 countries/regions are involved in nursing-related research on COVID-19, indicating that this field has gained considerable attention from researchers worldwide. In the list of the top 10 most productive countries, eight belong to developed countries and two (China and Turkey) belong to developing countries. Developed countries have higher GDP per capita than developing countries, and people living in developed countries typically emphasize their health and wellness. Furthermore, developed countries may invest more resources in defending against and investigating COVID-19. China, Iran, and India are the developing countries in Asia with large populations. Despite mobilization and allocation of resources to respond to the COVID-19 emergency, the high transmission rate of SARS-CoV-2, the large population base, the aging trend, and relatively scarce medical resources in these developing countries make combating COVID-19 a challenging task. Therefore, this study suggests that more nursing research related to COVID-19 should be conducted in these countries. Besides, among the most prolific authors and institutions, most came from the USA (8/15 authors and 5/10 institutions) and China (5/15 authors and 2/10 institutions). A previous study shows that the USA produced the most nursing-related funded publications from 2008 to 2018 (32). Therefore, it is deduced that the USA has dedicated a relatively huge sum of money, manpower, and material resources to nursing research during the COVID-19 pandemic. In addition, the USA also dominates nursing student education research (33) and Geriatric nursing research (34). However, most authors with the highest citation count came from China, and highly cited institutions are in China, followed by the UK and the USA. This can be explained by the fact that citations accumulate over time. Since the first case of COVID-19 was first reported at the end of 2019 in Wuhan, China, the country has received considerable attention worldwide, and articles published regarding the pandemic have gained a higher number of citations than that of other countries. Undoubtedly, researchers in China have offered an invaluable experience to worldwide readers in treating and fighting against the COVID-19 pandemic. In 2020, China declared the lockdown policy in Wuhan and managed to control the COVID-19 outbreak within 3 months, while many countries across Europe and the Americas struggled to contain the infection. This partly explains the increasing number of active authors and institutions besides those in China in 2021 and 2022. However, in 2022, the Omicron ripped off the “Zero policy” launched in China, and the third wave of COVID-19 hit many cities across the country. Nevertheless, there is a possibility that research in China will continue to be active again from 2022 onward.

### Journal Analysis

The top 10 prolific journals identified in this study are all ranked in higher quartiles in the category (Q1/Q2), according to the 2020 journal citation report (JCR). Besides the number of publications, CPP was also used to assess the quality of a journal. Among the top 10 prolific journals, only the Journal of Nursing Management (*n* = 12.2) and the Journal of the American Medical Directors Association (*n* = 11.2) have surpassed the average CPP of all publications (*n* = 10.48), while the remaining journals have gained fewer citations in this field. Thus, we list the top 10 co-cited journals, representing the most classical and influential journals in this field, such as the New England Journal of Medicine and the Lancet. The understanding of prolific journals could assist researchers in choosing the publisher for article submissions and grasping new topics. Also, publication in co-citation journals could add knowledge to the literature for future works (13).

### Hotspots and Research Trends

It is impossible to conduct a scientific investigation without acquiring prior knowledge. An analysis of keywords and references can provide maps of the knowledge and how it is interrelated (35, 36). In this study, using VOSviewer and CiteSpace, the knowledge structure of nursing-related research on COVID-19 is presented from the perspective of major keywords and classical references. The results demonstrate a gradual change in the topics investigated in this particular field. For example, in 2020, articles were centered on “SARS-CoV-2,” “knowledge,” “information teaching,” “mental health,” and “psychological problems,” while in 2021, the topics changed to “nursing student” and “vaccine-related issues”. The following section extends the keyword and citation analysis through cluster creation and briefly discusses it.

Cluster 1 represents mental health (green in Figure 7A). The primary keywords are “health care worker,” “nurse,” “mental health,” “anxiety,” “depression,” “stress,” and “burnout”. Nurses are a risk group for burnout syndrome because of the heavy caregiving responsibilities and the type of patients they care for (37). At the beginning of the COVID-19 pandemic, researchers focused on mental health problems in healthcare workers (Figure 6A). For example, the top two most-cited authors (Liu, ZC and Wang, Y) have focused on the impact of COVID-19 and associated factors on the mental health of healthcare workers in Wuhan, China. They reported that the intense work exhausts the healthcare workers working on the front line, physically and emotionally (8). The diagnosis of the healthcare providers can be broken down into 30% with insomnia symptoms, 40% with anxiety, 50% with depression, and 70% with distress (14, 30). Also, they emphasize that psychological assistance services (e.g., counseling, psychotherapy, mental health books, and tips on mental health self-help coping methods) are important for alleviating mental health disturbances (31). In addition, many scholars have compared the mental health problems between doctors and nurses. Shechter et al. (38) reported a higher rate of nurses experiencing COVID-19-related psychological distress (e.g., acute distress, anxiety, depression, and insomnia) than attending physicians in New York. Also, Giusti et al. (39) reported data from northern Italy, which informs the risks for emotional exhaustion and depersonalization during the COVID-19 pandemic were females working as a nurse in an emergency department or intensive care unit and in contacting COVID-19 patients. Hamularet et al. (40) reported that the higher prevalence of anxiety and level of hopelessness is higher in nurses than in other healthcare workers in Turkey. The reasons include fear of infection, lack of rest, inability to care for their children, emotion regulation difficulties, regret over the limitations of the visitation policy, and inability to provide adequate hospice care (41). Besides, the mental health of nursing students has also raised concerns among researchers. Savitsky et al. (11) analyzed the level of anxiety and associated factors of a cohort that involved 244 nursing students in Israel. They found that economic uncertainty, concern about infection, and difficulties in online learning are the key factors contributing to higher anxiety scores. Similarly, Gallego-Gomez et al. (42) reported that the homebound nursing students in Murcia (Spain) have a significantly high level of stress because of the 40-day lockdown, financial issues, family or emotional problems, less physical exercise, and failure to pass an online exam. Although most studies have focused on the adverse mental health effects of COVID-19, a few studies have investigated the factors that may mitigate these problems. Examples of mitigations include clear communication of commands and precautionary measures from organizations or employers and support, including provisions, adequate insurance, compensation, counseling, and psychological support (43). In addition, knowledge of control and coping strategy (42), relatively long work experience (more than 5 years), physical exercise (44), and social support (45) could also help decrease the level of psychological stress. Shechter et al. (38) conducted a web-based cross-sectional survey that involved 657 healthcare workers. They found that health workers have most frequently engaged in physical activity/exercise, followed by talk therapy to minimize the negative effects on mental health. However, the most effective method may be by providing nurses with adequate support, such as personal protective equipment, which should be the responsibility of the employers.

Cluster 2 represents nursing homes or long-term care (blue in Figure 7A). The primary keywords are “nursing home,” “older people,” “long-term care,” “mortality,” and “dementia.” Older people, especially those who live in nursing homes or long-term care facilities, are particularly at risk of contracting COVID-19 (26, 46). Grabowski et al. (47) reported that as of April 2020, nursing home residents and workers have contributed to about one-quarter of the recorded deaths caused by COVID-19 in the USA. Also, other countries such as the UK (48), Spain (49), and Canada (50) reported that residents of nursing homes have a higher rate of mortality. Some scholars have summarized the reasons that COVID-19 is more deadly to nursing home residents (51), which include the combination of the vulnerable elderly population, staffing shortages, inadequate resources, and a lack of effective treatments for COVID-19 patients (52). In addition, unlike community-dwelling older adults, nursing home residents could not obtain adequate social support and physical contact from their family, friends, and acquaintances during the mandatory lockdown and social isolation (53). Notably, the prevalence of dementia is higher in nursing home residents (47.8%) than the older adults living in the community (33%) (51). Older people with cognitive impairment rely almost exclusively on nursing home facilities to provide for their physical and psychological needs. Unfortunately, due to insufficient financial support, older adults with dementia have difficulty accessing high-quality psychological and emotional support from nursing homes, particularly those with low-quality ratings (54, 55). Thus, several scholars (56) have pointed out that the tragedy regarding nursing homes during COVID-19 resulted from decades of neglect of long-term care policy. This neglection takes several forms: lack of funding and monitoring institutions; insufficient training and underpaid staff; Medicare and Medicaid confusion and inadequacy in patient's home care, post-acute care, and long-time care; lack of small-scale, high-quality models that combine family care and long-term care in nursing homes. To address the crisis in nursing homes, the researchers have called for the consolidation of funding, policies, and new models that comprise institutional and non-institutional care (47, 56).

Cluster 3 represents nursing education (yellow in Figure 7A). The primary keywords are “nursing student,” “nursing education,” and “student”. COVID-19 has disrupted nursing students' education and clinical training. Nursing students could not undergo face-to-face clinical training due to inadequate equipment supplies, social isolation, and redeployment of clinical faculty members during the pandemic (57). However, studies have shown that online learning or virtual learning could be an effective substitute. Girao and her colleagues (58) developed a virtual reality game to train nursing students on how to prepare and administer medications; Weston et al. (59) developed a virtual clinical practice for pediatric nursing students; Luke et al. conducted a virtual exam for nursing students (60). Some studies have demonstrated the positive aspects of virtual learning: Herbert et al. (61) found their augmented reality app for remote training for heart failure could encourage nursing students to be more engaged in their learning process; Shamsaee et al. (62) reported that virtual education has significantly improved information-searching skills of nursing students. Luke et al. also reported that most nursing students and teaching staff have endorsed the interactive virtual clinical examination and considered it an effective alternative for training history-taking skills, communication, clinical decision-making, and patient management (60). However, researchers also expressed their concerns. Fitzgerald et al. (63) reported that in the first month after the COVID-19 outbreak, 90% of nursing students taking online courses experienced difficulty in concentrating, and 84% felt anxious or overwhelmed; Dutta et al. (64) revealed that 65% of Indian nursing students were dissatisfied with online study due to difficulty in interaction and focusing and lack of practical learning. Furthermore, virtual learning is also faced with challenges such as internet accessibility, difficulties with web conferences, inexperienced teachers, and a lack of motivation for students to learn online (65). Leighton et al. (66) compared the performance scores of 113 nursing students undergoing screen-based simulation learning, face-to-face simulation learning, and traditional clinical teaching. The study found that most traditional clinical teaching students scored higher than those undergoing screen-based simulation learning. The finding demonstrates the nature of nursing education as an applied discipline, which may not be teachable solely *via* a virtual learning model (67). Therefore, as virtual learning becomes more popular in nursing education, researchers should address the following issues in the future: (1) the need for teachers to pay attention to the mental health needs of nursing students; (2) the continuous improvement in online teaching competencies and experience, and conscious effort to encourage student–student, teacher–student, and student–computer interactions; (3) enhancement in the sense of immersion for better interactions, such as network synchronization, visual and haptic feedback, etc.; (4) combination of face-to-face and virtual learning during the COVID-19 pandemic and in future teaching activities.

Cluster 4 represents telemedicine (light blue in Figure 7A). The primary keywords are “telemedicine,” “telehealth,” and “primary care”. Before COVID-19, doctors and nurses already used telehealth tools (e.g., smartphones and related applications) in their daily work (68). However, the outbreak of COVID-19 has created a significant demand for telemedicine services, which provide continuity of primary care during social isolation, especially in chronic diseases (69) and cancer care (70). Sheba Medical Center rapidly shifted outpatient clinics to video consultations after the outbreak of COVID-19 in Israel (71). Gilkey et al. (72) reported that 89% of primary care providers in the USA used telehealth for their adolescent patients during COVID-19. There is evidence that care delivered *via* telemedicine is both safe and effective. For example, cancer patients have reported positive experiences with telemedicine, finding it convenient and acceptable for monitoring compliance and side effects of oral oncology treatments (73). Moreover, smartphones and applications allow doctors and nurses to repeatedly assess the adherence and symptoms of patients in real-time, thus improving the care of patients (74). However, despite the growing acceptance of “nursing telepractice,” challenges must be addressed. Barkai et al. (71) reported the results of a survey on satisfaction toward telemedicine during COVID-19, involving a cohort of 540 patients and 212 health workers. The study shows that 89.8% of patients have expressed satisfaction in contrast to only 37.7% of health workers who reported a high level of satisfaction. The study also reveals that 21% of patients and 80% of health workers have reported technical problems, and only 68% of health workers were willing to continue using telemedicine after the pandemic. Powell et al. (75) summarized the pitfalls of telemedicine during COVID-19 and provided future steps to improve the clinical application and convenience of telehealth services, which are as follows: (1) what kind of consultation is best suited for a particular category of a patient?; (2) how can telemedicine be made easier to be used and customized?; (3) how can the benefits of telehealth be maximized for doctors and nurses and support their use of telehealth in future?; (4) what skills should nurses acquire to provide better telehealth services?

Cluster 5 represents vaccine and infection control (yellow and purple in Figure 7A). The primary keywords are “vaccination,” “vaccine,” “vaccine hesitancy,” “public health,” and “infection control”. Vaccination-related issues have received increasing attention from researchers and the public (76). People in different countries have expressed their vaccine hesitancy due to the fear of side effects and lack of confidence in the effectiveness of the vaccine (77). Healthcare workers are standing on the frontline against COVID-19, and vaccination is one of the key measures to protect them (78). Thus, scholars have investigated the intention to acquire vaccination among nurses and health workers. For example, Trabucco et al. (79) conducted an online survey to predict the level of acceptance toward the upcoming COVID-19 vaccine in Italy. They found that 91.5% (486/531) of nurses were willing to accept the vaccine, whereas 2.3% were against the vaccination program. In Cyprus, Fakonti et al. (80) reported that 70% (306/4377) of nurses and midwives did not intend to receive the COVID-19 vaccine, while Sun et al. (81) reported that 25% of nurses refused to accept the vaccine in China. Although the vaccine acceptance rates in different countries or regions are different, most cited reasons revolve around the fear of side effects, having no knowledge of the type of vaccine, distrust in the vaccine's effectiveness, female, and concerns over rapid mutation in the virus (82). In particular, the rapid mutation in the virus has raised concerns regarding the possibility of population-wide vaccination to contain COVID-19. However, vaccination is believed to be the best method to reduce COVID-19-related mortality at present (83). Notably, nurses should assume the role of a trustworthy and credible source of vaccine-related information to build public confidence in vaccination programs. Thus, policymakers should mitigate the COVID-19 vaccine-related side effects and build public confidence in vaccination programs to enhance the vaccination rate and control the spread of infection.

### Limitations

There are several limitations identified in this study. First, to comply with the data format for bibliometric tools in both VOSviewer and CiteSpace, the nursing-related research on COVID-19 was collected from a single database (WOSCC), which might have resulted in selection bias. There are other data sources, such as PubMed or Scopus, but most are only compatible with either one of the bibliometric tools, frequently the VOSviewer. Therefore, we opted for using two bibliometric tools (CiteSpace and VOSviewer) to reduce selection bias and eliminate the inconvenience of integrating duplicate literature from multiple databases. Second, this study used only the number of publications, TC, and CPP as indicators for the quality of a paper, author, or institution. Other metrics (e.g., H-index, Impact Factor, CiteScore) are also accepted by researchers (84). Third, this study might be limited by language bias because only articles published in English were included. Future research should incorporate publications in other languages to obtain comprehensive results. Last, as of this writing, this study only included articles published up to the first 6 months of 2022, and thus, further updates in WoSCC may change the ranking of authors, institutions, etc., presented in this study. However, we believe that the low citation frequency of newly published articles produces little impact on our main findings.

## Conclusion

This bibliographic analysis provides an overview of nursing-related research on COVID-19. During the COVID-19 pandemic, topics such as “mental health,” “telemedicine,” “nursing education,” “nursing home,” and “vaccine-related issues” have attracted considerable attention. Further work should emphasize the following initiatives: (1) providing nurses with adequate support to reduce psychological stress, especially from employers or organizations; (2) increasing investments to change long-term care policies in nursing homes: (3) combining face-to-face teaching and virtual learning in nursing education; (4) improving clinical and administrative applications of telemedicine services; (5) reducing the COVID-19 vaccine-related side effects and building up public confidence in vaccination programs.

## Data Availability Statement

The original contributions presented in the study are included in the article Supplementary Material, further inquiries can be directed to the corresponding author/s.

## Author Contributions

QZ and SL conceived of the study, participated in its design, and drafted the manuscript. JL and SL were involved in the study design, obtained data and contributed to interpretation, and helped to draft the manuscript. JC provided the theoretical frameworks and performed much of the editing of the manuscript. SL helped a lot in the revision process, collecting data, organizing literature and redo the figures and tables. All authors contributed to the article and approved the submitted version.

## Funding

This study was supported by the Hunan Science and Technology Innovation Platform and Talent Plan [Grant: 2017TP1004] and Scientific research project of Guangxi Education Department (2020KY12036).

## Conflict of Interest

The authors declare that the research was conducted in the absence of any commercial or financial relationships that could be construed as a potential conflict of interest.

## Publisher's Note

All claims expressed in this article are solely those of the authors and do not necessarily represent those of their affiliated organizations, or those of the publisher, the editors and the reviewers. Any product that may be evaluated in this article, or claim that may be made by its manufacturer, is not guaranteed or endorsed by the publisher.
