# Supplementary material for: Global Trends in Nursing-Related Research on COVID-19: A Bibliometric Analysis
Source: Front Public Health. 2022 Jul 18;10:933555. doi: 10.3389/fpubh.2022.933555 (PMC9339968; doi:10.3389/fpubh.2022.933555)
Supplement: Supplementary file 1 [file Table_1.pdf]

Table S1. Thesaurus

label	replace by
attitudes	attitude
coronavirus disease 2019	covid-19
coronavirus disease 2019 (covid-19)	covid-19
covid	covid-19
covid 19	covid-19
covid-19 pandemic	covid-19
health care workers	health care worker
health personnel	health care worker
health professionals	health care worker
health workers	health care worker
healthcare	health care worker
healthcare personnel	health care worker
healthcare professionals	health care worker
healthcare worker	health care worker
healthcare workers	health care worker
health-care workers	health care worker
hospitals	hospital
infectious diseases	infectious disease
intensive care units	intensive care unit
medical staff	health care worker
nurses	nurse
nursing homes	nursing home
nursing students	nursing student
elderly	older people
older adults	older people
pandemics	pandemic
